# Specific RNA structures and elements in the 5′-UTR of the SARS-CoV-2 genome and subgenomic RNA are critical for its infection

**DOI:** 10.1016/j.gendis.2026.102030

**Published:** 2026-01-07

**Authors:** Yan Zhan, Xi Li, Ke Liu, Qian-Ying Ouyang, Jia-Jia Cui, Chen-Hui Luo, Ji-Ye Yin

**Affiliations:** a Department of Clinical Pharmacology, Xiangya Hospital, Central South University, Changsha, Hunan 410078, China; b Engineering Research Center of Applied Technology of Pharmacogenomics, Ministry of Education, Changsha, Hunan 410078, China; c National Clinical Research Center for Geriatric Disorders, Changsha, Hunan 410008, China; d Department of Pharmacy, Xiangya Hospital, Central South University, Changsha, Hunan 410008, China; e Research Core Facility, Hunan Cancer Hospital, The Affiliated Cancer Hospital of Xiangya School of Medicine, Central South University, Changsha, Hunan 410013, China; f Department of Lymphoma & Hematology, Hunan Cancer Hospital, The Affiliated Cancer Hospital of Xiangya School of Medicine, Central South University, Changsha, Hunan 410013, China; g Department of Geriatric Surgery, Xiangya Hospital, Central South University, Changsha, Hunan 410008, China; h Department of General Surgery, Xiangya Hospital, Central South University, Changsha, Hunan 410008, China

Since its emergence in December 2019, severe acute respiratory syndrome coronavirus 2 (SARS-CoV-2) has caused hundreds of millions of infections and deaths worldwide, posing a considerable threat to human health. SARS-CoV-2 belongs to the β-coronavirus family and displays a typical genome structure.^1^ Upon entering a host cell, the virus initiates replication of its genomic RNA and subsequently generates nine subgenomic RNAs.^2^ These RNA molecules are utilized to produce various viral proteins within the host cell, ultimately leading to the assembly of new viral particles. Thus, the translation of SARS-CoV-2-encoded mRNAs is fundamental to the formation of viral particles that can infect the human host.^3^ Published studies have shown that the 5′-untranslated region (UTR) is critical for translational control of SARS-CoV-2 viral proteins, especially translation initiation.^4^^,^^5^ However, the exact regulatory strategies and translation elements involved have not been well explored. In this study, we employed a combination of experimental and bioinformatics approaches to identify specific RNA structures and elements within the 5′-UTR of the SARS-CoV-2 genome and its subgenomic RNAs that are critical for viral infection.

The 5′-UTR sequences of the SARS-CoV-2 genomic RNA and nine subgenomic RNAs (ORF3a, ORF6, ORF7a, ORF7b, ORF8, N, S, M, E) were obtained from the UCSC database (https://genome.ucsc.edu/). The lengths of viral 5′-UTRs range from 75 nt to 265 nt (Table S1), and all contain a 75 nt leader sequence (Fig. S1A). To assess the capability of viral 5′-UTRs in regulating gene expression, we cloned these sequences into the PGL3-promoter vector and constructed a series of new plasmids for dual-luciferase reporter gene assays (Table S4; Fig. S1B). The experimental results demonstrate a significant increase in luciferase expression driven by the 5′-UTRs of both SARS-CoV-2 gRNA and sgRNAs compared to the control group (Fig. S1C–S1J), indicating that the 5′-UTR plays an important role in the process of stable expression of viral proteins in large quantities. Notably, changes in the length of 5′-UTRs are not completely proportional to the translation regulation ability. Therefore, we speculated that there are specific translation regulatory factors within different 5′-UTRs.

The secondary structure of the 5′-UTR in mRNA plays a critical role in regulating ribosome recruitment and the scanning mechanism for the start codon, thereby modulating translation efficiency. As an initial step, we predicted the RNA secondary structures for all 5′-UTRs using the RNAfold server (Fig. S2A–S2H). Analysis of GC content revealed that the 5′-UTRs of gRNA (44.53%) and ORF6-sgRNA (40.87%) possess relatively high GC content, which suggests that these regions form highly stable secondary structures. Furthermore, a clear difference in GC content was observed between the 5′-UTRs of gRNAs and sgRNAs. The gRNA and ORF6 5′-UTRs exhibited low free energy, with minimum free energy (MFE) values below −50 kcal/mol, indicating highly stable secondary structures. Given that an MFE below this threshold inhibits cap-dependent translation, our findings suggest that these viral RNAs may utilize a cap-independent translation mechanism (Fig. 1A).

**Figure 1:**
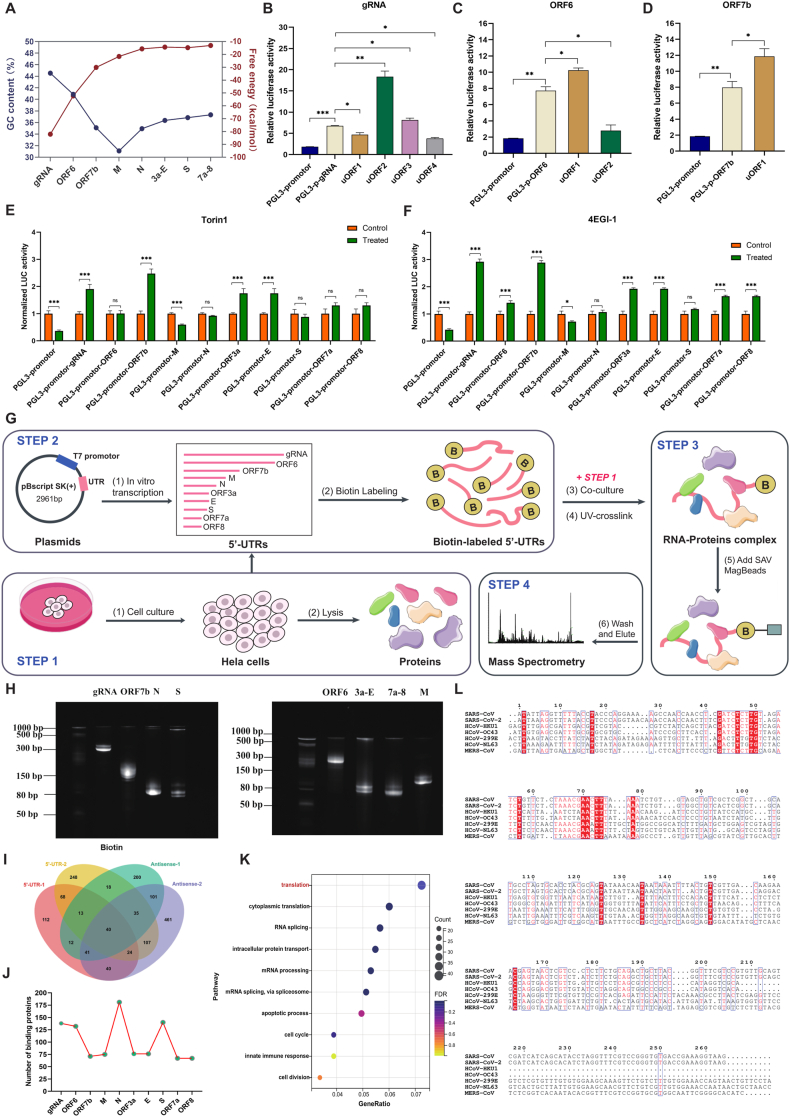
The role of 5′-UTR RNA structures and functional elements in SARS-CoV-2 infection. **(A)** Free energy (red line) and GC content (blue line) of 5′-UTRs. Since the 5′-UTR sequences of ORF3a and E are identical, we use “3a-E” to represent their common secondary structure in the results, and “7a-8” (ORF7a and ORF8) follows the same naming rules; RNA secondary structures of genomic and subgenomic 5′-UTRs are predicted by RNAfold. **(B**–**D)** Relative luciferase activities of mutant constructs in HeLa cells: (B) gRNA, (C) ORF6, and (D) ORF7b. **(E)** Normalized luciferase activity of the PGL3-promotor-5′-UTR construct under Torin1 treatment (9 nM). **(F)** Normalized luciferase activity of the PGL3-promotor-5′-UTR construct under 4EGI-1 treatment (25 μM). Data are presented as means ± standard deviation and are representative of the results from at least three independent experiments. Statistical significance was calculated via Student's *t*-test. **(G)** Outline of the BPD-MS method used to identify proteins bound to the SARS-CoV-2 genomic and subgenomic 5′-UTRs. **(H)** Urea-PAGE gel validates the 5′-UTRs produced by *in vitro* transcription. **(I)** Venn diagram of biotin-labeled 5′-UTRs-binding protein and biotin-labeled antisense RNA-binding protein. **(J)** Number of proteins bound to SARS-CoV-2 5′-UTRs. **(K)** Gene Ontology analysis of proteins significantly regulated in global proteome measurements. **(L)** Conservation analysis of the 5′-UTRs of SARS-CoV-2 and six other human-infecting coronaviruses.

In addition to RNA secondary structure, several elements within the 5′-UTR, including RNA G-quadruplexes, internal ribosome entry sites (IRES), and upstream open reading frames (uORFs), play crucial roles in translational regulation. Although G-quadruplexes are involved in transcriptional and translational control across species, none were predicted in the SARS-CoV-2 5′-UTRs using the QGRS mapper. Conversely, IRES elements, which facilitate cap-independent ribosome recruitment, were predicted via IRESPred and IRESite (Table S2) in the 5′-UTRs of gRNA and partial sgRNAs (ORF6, ORF7b, and M). Notably, the IRES in the M-sgRNA was specifically located between nucleotides 76 and 103. Furthermore, eight uORFs were identified across these UTRs using ORFfinder (Fig. S3A). Mutational disruption of their start codons revealed divergent effects on translation (Table S6): mutation in gRNA-uORF2, gRNA-uORF3, ORF6-uORF1, or ORF7b-uORF1 increased downstream translation, suggesting inhibitory roles. In contrast, mutating gRNA-uORF1, gRNA-uORF4, or ORF6-uORF2 reduced translation, indicating their enhancing functions (Fig. 1B–D). These findings imply that the 5′-UTRs of SARS-CoV-2 genomic and subgenomic RNAs may employ IRES-mediated mechanisms and context-dependent uORFs to finely regulate viral protein synthesis.

Based on previous evidence suggesting that SARS-CoV-2 may utilize cap-independent translation mechanisms, we sought to investigate this further. We inhibited cap-dependent initiation using Torin1, an mTOR inhibitor that suppresses 4E-BP phosphorylation. Treatment with Torin1 (9 nM, 24 h) significantly enhanced luciferase activity under the control of 5′-UTRs from gRNA (1.9-fold), ORF7b- (2.47-fold), ORF3a- (1.75-fold), and E-sgRNA (1.75-fold), indicating robust cap-independent translation (Fig. 1E). Further inhibition with 4EGI-1, which disrupts eIF4E/eIF4G interaction, reduced M-sgRNA translation to 70% of the control, confirming its cap-dependent preference. In contrast, translation from gRNA and multiple sgRNAs (ORF6, ORF7b, ORF3a, E, ORF7a, ORF8) increased markedly (up to 2.91-fold), supporting cap-independent initiation. The 5′-UTRs of N and S exhibited no significant change, suggesting mixed mechanisms (Fig. 1F). These results demonstrate widespread cap-independent translation among SARS-CoV-2 genomic and subgenomic RNAs.

Being obligate intracellular parasites, viruses rely crucially on host cells for survival and are incapable of independent metabolism or replication outside a host. SARS-CoV-2 extensively hijacks host cellular machinery throughout its replication cycle. To elucidate the role of host proteins in facilitating viral component synthesis, we investigated the interactions between viral 5′-UTRs and host proteins (Fig. 1G). Through *in vitro* transcription and Urea-PAGE validation, we obtained 5′-UTRs from the SARS-CoV-2 genome and subgenomic RNAs (Fig. 1H; Table S5). Biotin-based RNA pull-down assays coupled with LC–MS/MS were used to capture and identify host proteins bound to these regions, with antisense and non-biotinylated controls included to ensure specificity and minimize non-specific binding (Fig. 1I). We identified varying numbers of proteins associated with each 5′-UTR: gRNA (138), ORF6 (132), S (140), N (181), ORF7b (71), M (75), ORF3a (76), E (76), ORF7a (67), and ORF8 (67). Generally, binding protein counts correlated with UTR length, though N- and S- 5′-UTRs bound markedly more proteins than expected based on length alone (Fig. 1J). A subset of bound proteins (2%–10%) remains uncharacterized (Fig. S3B). Gene Ontology analysis indicated significant enrichment of translation-related functions among the captured proteins (Fig. 1K; Fig. S3C–S3J), including ribosomal subunits, translation initiation/elongation factors, poly A-binding proteins, and m^6^A readers (Fig. S4). These findings suggest both cap-dependent and cap-independent translation mechanisms are exploited by SARS-CoV-2. Protein–RNA interaction networks further revealed that the 5′-UTRs recruit host proteins involved not only in translation but also in RNA processing, splicing, and protein transport (Fig. S5). This indicates a broader hijacking of host machinery to support viral replication, protein synthesis, and transport.

Due to the high transmissibility of SARS-CoV-2, we compared its 5′-UTR with those of six other human-infecting coronaviruses (Table S3). Phylogenetic analysis confirmed low similarity across genera (Fig. 1L). We identified 39 highly conserved nucleotides (≥ 85% occupancy), primarily within positions 1–196. The longest consecutive conserved tract was 3 nt, located in the 1–75 nt leader region, suggesting potential functional importance of these elements across coronaviruses.

In conclusion, we found that the SARS-CoV-2 genome and subgenomic 5′-UTRs can promote translation, and further exploration revealed the structural characteristics and regulatory elements of the 5′-UTRs. A network of RNA 5′-UTR-human host protein interactions was established. These findings suggest that the 5′-UTR regulates viral protein translation and is responsible for the massive replication of SARS-CoV-2. Furthermore, the current study may change the direction of disease treatment and promote the development of RNA vaccines targeting UTR regions to control emerging infectious diseases and future pandemics.

## CRediT authorship contribution statement

**Yan Zhan:** Writing – review & editing, Writing – original draft, Visualization, Validation, Project administration, Methodology, Investigation, Formal analysis, Data curation, Conceptualization, Software. **Xi Li:** Visualization, Validation, Formal analysis, Data curation, Software. **Ke Liu:** Formal analysis, Software, Validation. **Qian-Ying Ouyang:** Formal analysis, Software. **Jia-Jia Cui:** Supervision, Project administration, Methodology, Funding acquisition, Conceptualization, Validation, Writing – original draft. **Chen-Hui Luo:** Conceptualization, Funding acquisition, Methodology, Project administration, Supervision, Writing – original draft, Writing – review & editing. **Ji-Ye Yin:** Writing – review & editing, Validation, Supervision, Project administration, Investigation, Funding acquisition, Conceptualization, Methodology.

## Funding

This work was supported by the 10.13039/501100001809National Natural Science Foundation of China (No. 82373962, 82450103, 82204533), the Scientific Research Project of Furong Laboratory of Central South University (China) (No. 2023SK2083), the 10.13039/501100004735Natural Science Foundation of Hunan Province of China (No. 2023JJ40931), the Open Fund of Shenzhen Key Laboratory of Chinese Medicine Active Substance Screening and Translational Research (China) (No. ZDSYS20220606100801003), High-Level Talent Support Program of Hunan Cancer Hospital; Hunan Cancer Hospital Climb Plan (China) (No. YF2020011) and the Scientific and Technological Project of Hunan Province (China) (No. 2023ZJ1123).

## Conflict of interests

The authors declare that they have no conflict of interests.
