# Supplementary material for: Specific RNA structures and elements in the 5′-UTR of the SARS-CoV-2 genome and subgenomic RNA are critical for its infection
Source: Genes Dis. 2026 Jan 7;13(6):102030. doi: 10.1016/j.gendis.2026.102030 (PMC13380148; doi:10.1016/j.gendis.2026.102030)
Supplement: Multimedia component 1 [file mmc1.docx]

**Materials and methods**

**Plasmids**

All 5'-UTR sequences of gRNA and sgRNAs were obtained from the UCSC Genome Browser (<https://genome.ucsc.edu/>). The 5'-UTRs were subsequently cloned and inserted into the PGL3-promoter vector between the HindIII and NcoI restriction sites (Table S4). The constructs were delegated to Tsingke Biotech (Beijing, China). The PGL3-promoter empty plasmid was purchased from Tsingke Biotech (Beijing, China). The reverse complements of the 5'-UTRs were introduced into the pBluescript II SK(+) vector between the EcoRI and BamHI restriction sites (Table S5). The constructs were sent to Sangon Biotech (Shanghai, China). The mutant PGL3-promoter-gRNA, PGL3-promoter-ORF6, and PGL3-promoter-ORF7b plasmids were generated via site-directed mutagenesis via the Mut Express II Fast Mutagenesis Kit V2 (Vazyme, Nanjing, China) according to the manufacturer's instructions, and the primer sequences are shown in Table S6. All the constructed plasmids were verified via Sanger sequencing.

**Cell culture**

The HeLa cell line was purchased from the Cell Bank of the Chinese Academy of Sciences (Shanghai, China). The cells were grown in RPMI-1640 medium (Gibco, New York, USA) supplemented with 10% fetal bovine serum (Gibco, New York, USA) and 1% penicillin/streptomycin (Gibco, New York, USA) in 5% CO_2_-buffered incubators at 37°C. The cells were subcultured at regular intervals whenever the confluence reached 80-90%.

**Dual-****luciferase reporter gene assays**

HeLa cells were seeded in 24-well plates at a density of 1×10^5^ cells/well and grown to 60-70% confluence. Plasmid DNA was prepared with an Endo-Free Plasmid Mini Kit I (Omega Biotek, Georgia, USA). HeLa cells were transfected with Lipofectamine 3000 Reagent (Invitrogen, California, USA) according to the manufacturer's instructions. pRL-TK (Beyotime, Shanghai, China) was cotransfected as an internal control to normalize the transfection efficacy. After 6 h of transfection, the cells were treated with Torin1 (9 nM) (MedChemExpress, New Jersey, USA)/4EGI-1 (25 μM) (MedChemExpress, New Jersey, USA) or 0.1% DMSO (vehicle control, Sigma, Missouri, USA). After 24 h of treatment, the cells were rinsed twice with PBS (pH 7.4) and lysed with 1× passive lysis buffer (100 μL/well). Luminescence was measured via the Dual-Luciferase Reporter Assay System (Promega, Wisconsin, USA) following the manufacturer's instructions. The mutant plasmid was not treated with chemicals. After 6 hours of transfection, the culture medium was replaced with fresh medium, and the cells were harvested for luminescence detection after 24 hours.

**In vitro RNA transcription**

The restriction endonucleases BamHI (New England Biolabs, Massachusetts, USA) or ECORI (New England Biolabs, Massachusetts, USA) were used to digest pBluescript II SK(+) series plasmids, followed by 1% agarose gel electrophoresis and gel purification (QIAquick Gel Extraction Kit, QIAGEN, Hilden, Germany) to obtain linearized template DNA for in vitro transcription. The MEGAscript T7 Transcription Kit (Invitrogen, California, USA) or the MEGAscript T3 Transcription Kit (Invitrogen, California, USA) were used to generate sense 5'-UTRs or antisense 5'-UTRs, respectively. An RNA Clean & Concentrator Kit (Zymo Research, California, USA) was used to purify the RNA. A 5% acrylamide/8 M urea gel was used to verify the purity of the RNA, and the RNA marker used was the Century-Plus RNA marker (Invitrogen, California, USA). Finally, biotin labeling was performed on RNA transcribed in vitro by T7 and T3 RNA polymerases (10x Biotin RNA labeling mix; Roche, Basel, Switzerland). The above reagents were used according to the manufacturer's instructions.

**Biotin-pull down assays**

Biotin-labeled sense and antisense 5'-UTRs were prepared in previous experiments. The HeLa cell lysates were mixed with the biotinylated sense 5'-UTRs and incubated at approximately 25 °C for 1 h, and the biotinylated antisense 5'-UTRs served as a negative control. The streptavidin magnetic beads (Invitrogen, California, USA) were mixed with the RNA mixture at room temperature for 30 minutes, and the biotin-coupled RNA complexes were eluted and used for mass spectrometry analysis.

**Mass spectrometry analysis for the 5**'**-UTR-interactome**

Protein digestion was performed via the filter-aided sample preparation method [25]. The detergents DTT and IAA in the UA buffer were added to block reduced cysteine. The protein suspension was digested with 2 µg of trypsin (Promega, Wisconsin, USA) overnight at 37 °C. The peptides were collected by centrifugation at 16000 × g for 15 min and desalted with C18 StageTip for further LC-MS analysis.

LC-MS/MS experiments were performed on a Q Exactive Plus mass spectrometer that was coupled to an Easy nLC1200 instrument (Thermo Scientific) by Shanghai Bioprofile Technology Company Ltd. (Shanghai, China). MS data were acquired via a data-dependent top-20 method in which the most abundant precursor ions from the survey scan (350-1800 m/z) were dynamically chosen for higher-energy collision dissociation fragmentation. A lock mass of 445.120025 Da was used as an internal standard for mass calibration. The full MS scans were acquired at resolutions of 70,000 at m/z 200 and 15,000 at m/z 200 for the LC-MS/MS scan. The maximum injection time was set to 50 ms for MS and 25 ms for LC-MS/MS. The normalized collision energy was 28, and the isolation window was set to 1.6 Th. The dynamic exclusion duration was 30 s. The MS data were analyzed via MaxQuant software version 1.6.1.0. The database search results were filtered and exported with a <1% false discovery rate at the peptide-spectrum-matched level and protein level.

**Bioinformatics analysis and visualization**

The secondary structures of the genomic and subgenomic 5'-UTRs were predicted via RNAfold [26]. The free energy of the secondary structures was calculated via RNAfold (<http://rna.tbi.univie.ac.at/cgi-bin/RNAWebSuite/RNAfold.cgi>) with the default settings. The presence of potential G-quadruplex structures was indicated by the QGRS Mapper [27]. The IRESs were predicted by IRESite [28] and IRESPred [29] (<http://196.1.114.46:1800/IRESPred/home.html>) with default settings. All the uORFs located in the 5'-UTRs were discovered via NCBI ORFfinder [30] (<https://www.ncbi.nlm.nih.gov/orffinder/>). The DAVID website (<https://david.ncifcrf.gov/>) was used to perform GO analysis, and P values <0.05 were considered statistically significant. Bar graphs and line graphs were drawn via GraphPad Prism 9 (version 9.0.0). The network diagram and Venn diagram were drawn with Cytoscape (version 3.7.1) and Sangerbox (<http://vip.sangerbox.com/home.html>), respectively. MEGA software (version 7.0.26) was used for conservation analysis, the algorithm was ClusterW, and the sequence alignment results were finally visualized on the ENDscript/ESPript website (<https://espript.ibcp.fr/ESPript/cgi-bin/ESPript.cgi>).

**Statistical analysis**

All the data are presented as the means of three or more independent experiments, and the error bars indicate the standard deviations of the means. Statistical analysis was performed via GraphPad Prism 9 (version 9.0.0). The significance symbols for all the experiments are as follows: ns *P* > 0.05; * *P* < 0.05; ** *P* < 0.01; *** *P* < 0.001.
