# Supplementary material for: Specific RNA structures and elements in the 5′-UTR of the SARS-CoV-2 genome and subgenomic RNA are critical for its infection
Source: Genes Dis. 2026 Jan 7;13(6):102030. doi: 10.1016/j.gendis.2026.102030 (PMC13380148; doi:10.1016/j.gendis.2026.102030)

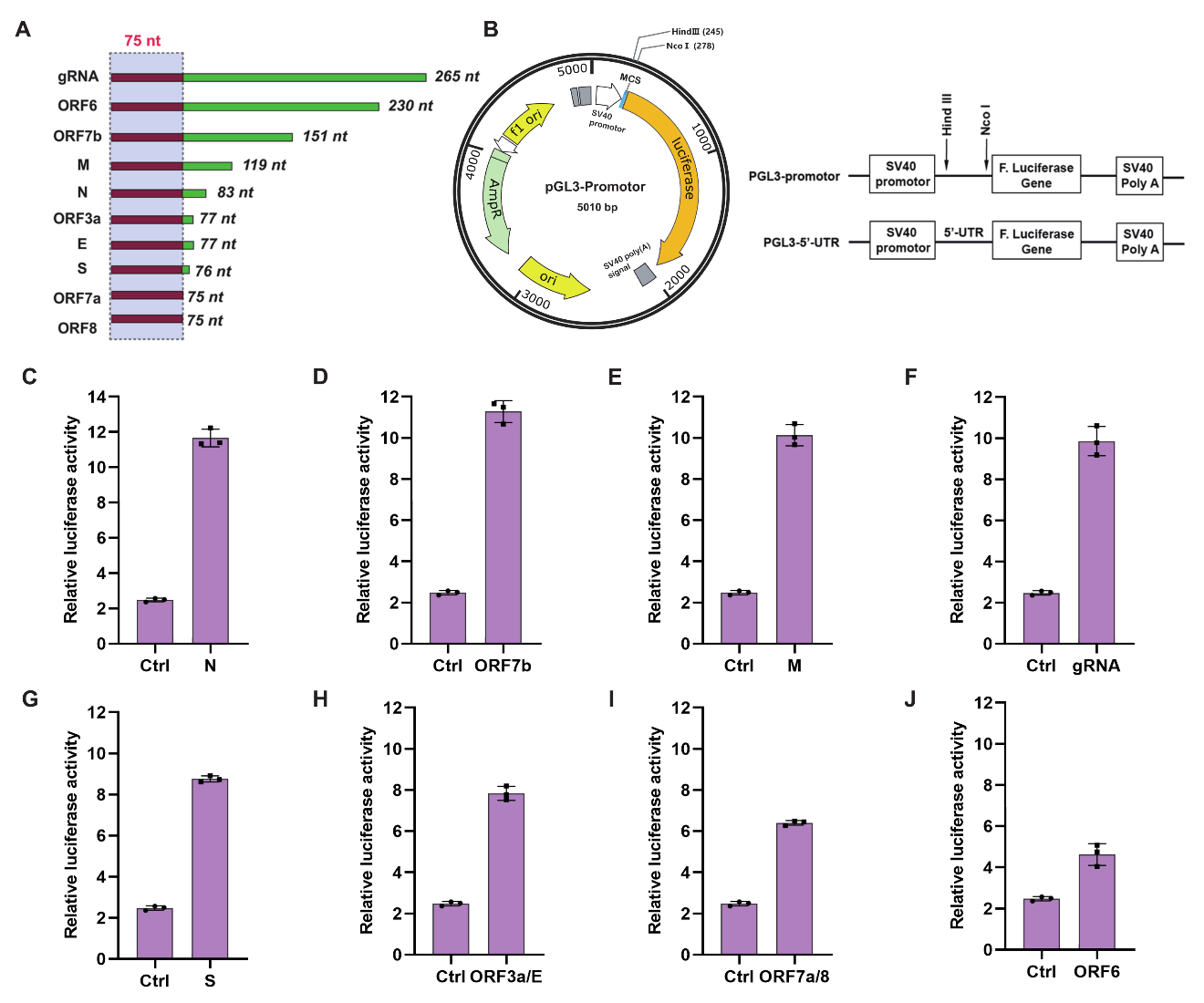
**Figure S1** The 5'-UTRs of the SARS-CoV-2 genome and subgenomes promote translation of its downstream sequence in HeLa cells. (A) Simplified diagram of the location and length of 5'-UTRs in genomic and subgenomic RNAs. (B) schematic diagram of the construction of PGL3-promoter-5'-UTR series plasmids. Relative LUC activities generated by the PGL3-promoter-5'UTR constructs in HeLa cells: (C) N, (D) ORF7b, (E) M, (F) gRNA, (G) S, (H) ORF3a/E, (I) ORF7a/8 and (J) ORF6. The data are presented as the means ± SD and are representative of the results of at least 3 independent experiments.


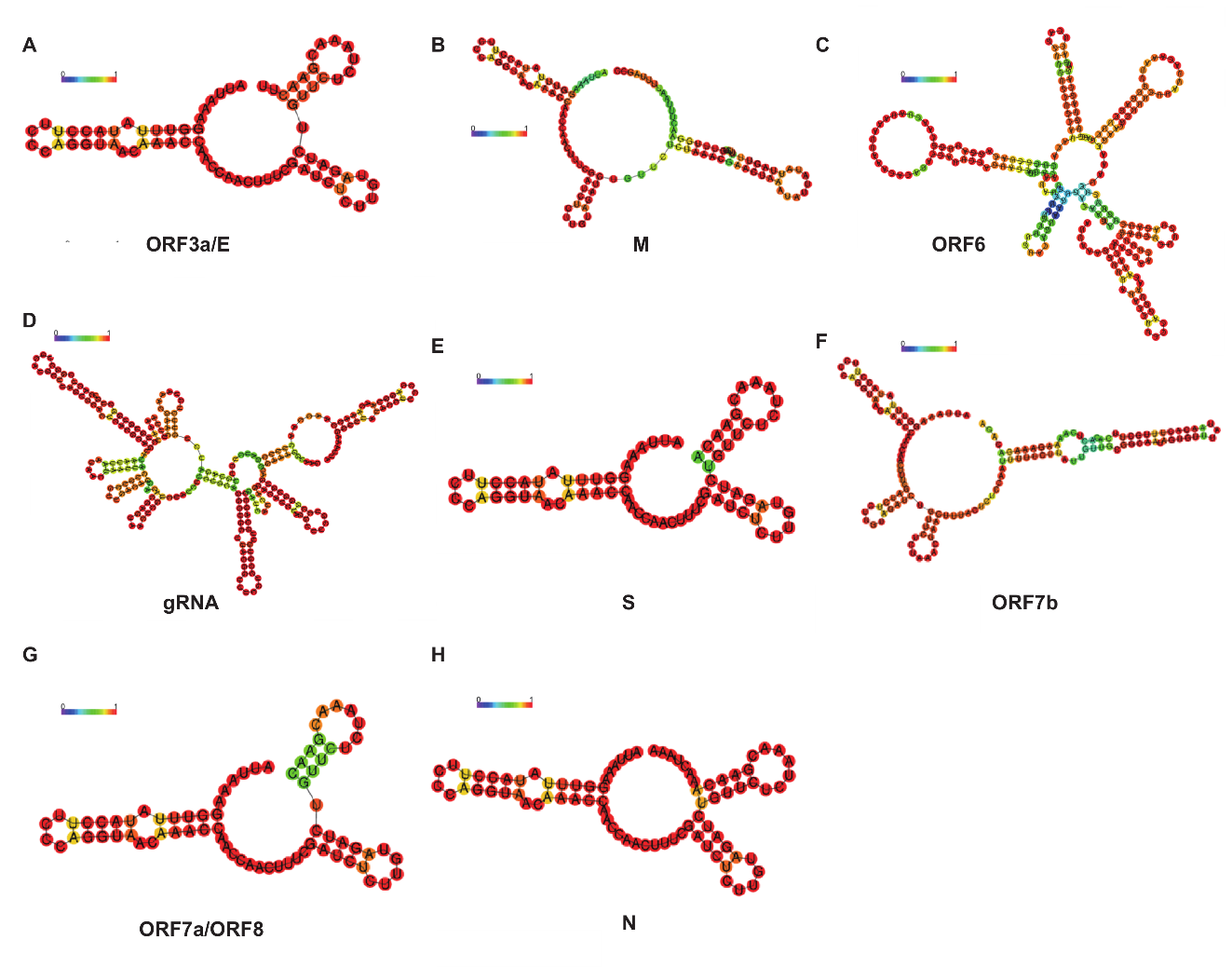


**Figure S2** RNAfold-predicted RNA secondary structures of genomic and subgenomic 5'-UTRs. RNA secondary structure of (A) ORF3a/E, (B) M, (C) ORF6, (D) gRNA, (E) S, (F) ORF7b, (G) ORF7a/ORF8, and (H) N.

**
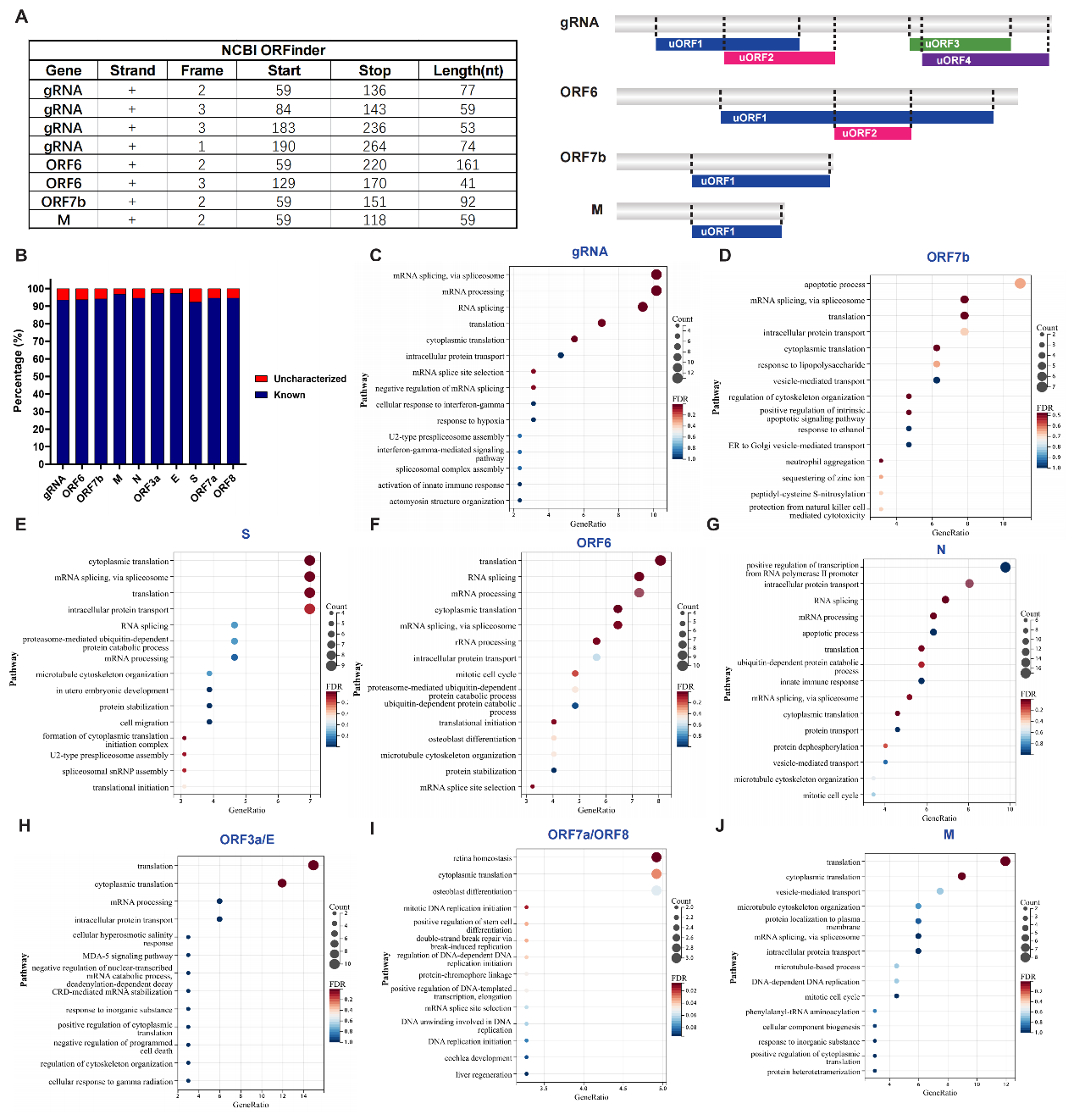
**

**Figure S3** Predicted uORFs and identified host proteins bound to SARS-CoV-2 5'-UTRs. (A) uORFs predicted by ORFfinder. The left panel shows the prediction results for the uORFs, and the right panel shows a simplified map of the uORF locations. (B) Proportion of proteins with undetermined functions among proteins bound to each RNA. (C-J) Gene ontology analysis of host proteins bound by SARS-CoV-2 genomic and subgenomic 5'-UTRs.


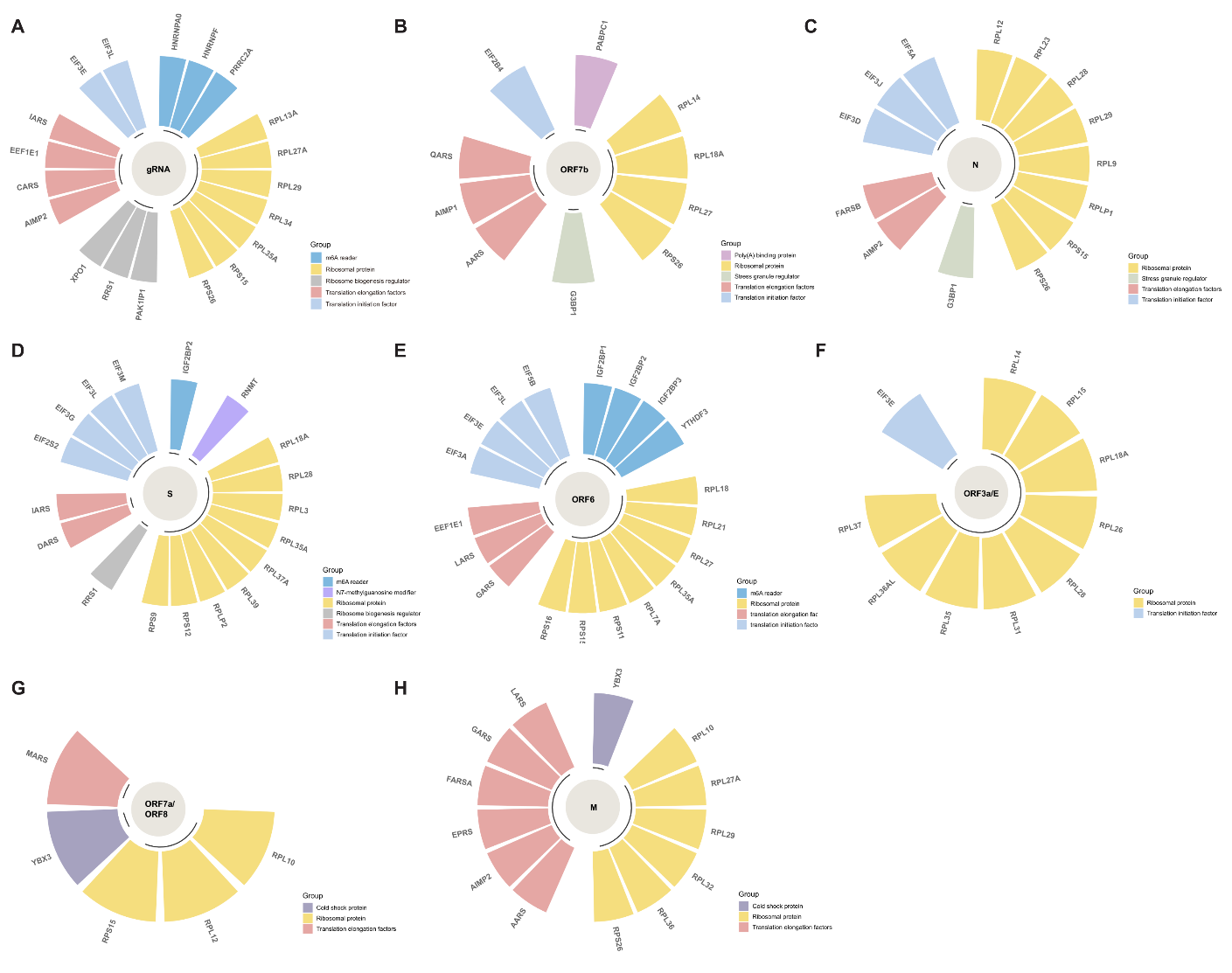


**Figure S4** A detailed analysis of the translation-related proteins bound by SARS-CoV-2 genomic and subgenomic RNA 5'-UTRs. (A) gRNA; (B) ORF7b; (C) N; (D) S; (E) ORF6; (F) ORF3a/E; (G) ORF7a/ORF8; (H) M. Different colors represent different kinds of proteins.

**Figure S5** Schematic of the RNA-protein interaction network of 5'-UTR SARS-CoV-2 RNAs. Orange rhombi, 5'-UTRs; oval pattern, host protein. The interacting host protein and the 5'-UTR are connected with a line, and the thickness of the line represents the intensity. Proteins belonging to the top 10 pathways are marked in bright colors, and the remaining proteins are marked in gray.
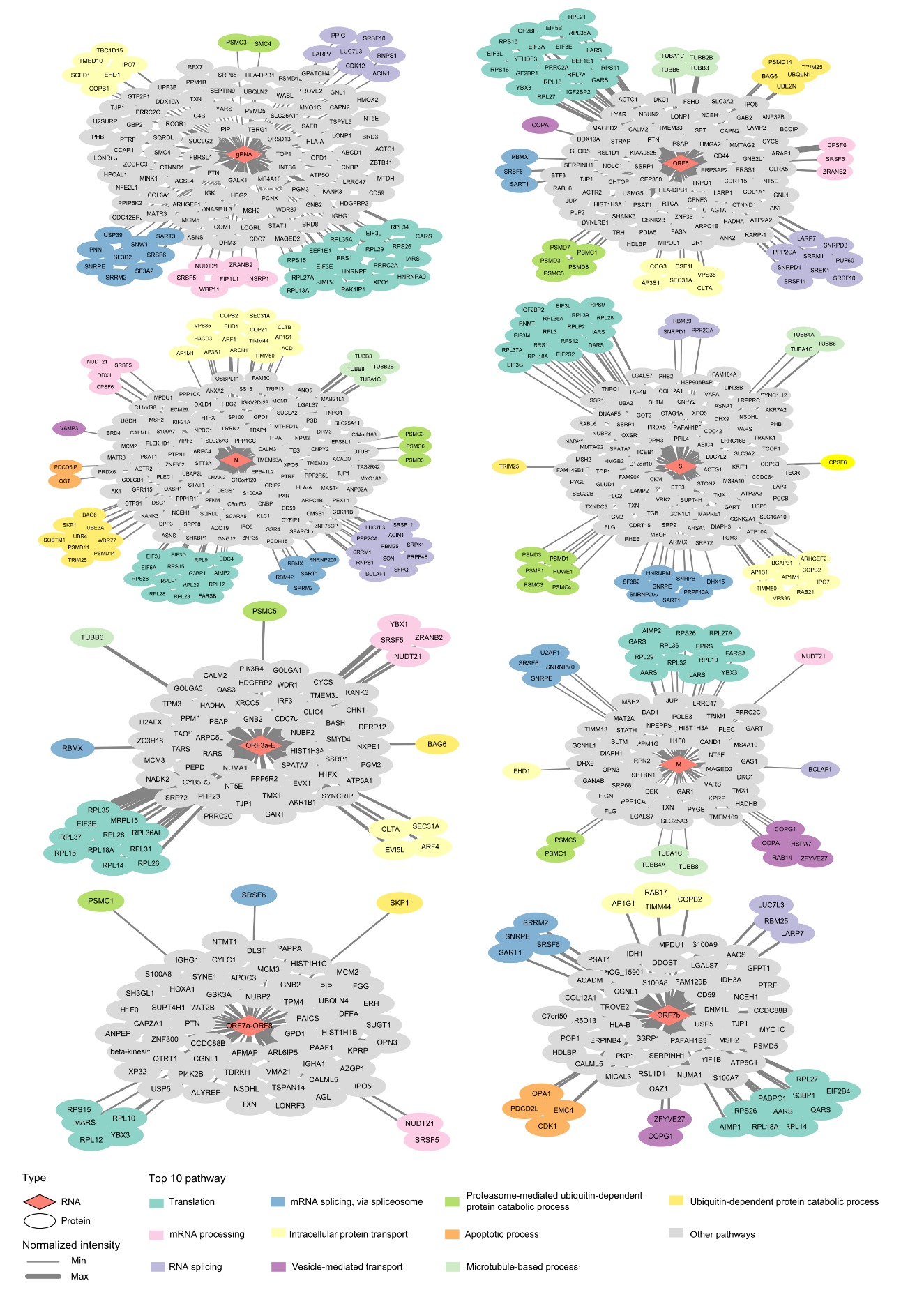

Supplement: Multimedia component 2 [file mmc2.docx]
